# Knowledge Acquisition in Times of the 2020 Coronavirus Pandemic: Evidence from a Four-Wave Panel Study

**DOI:** 10.1093/ijpor/edab017

**Published:** 2021-08-30

**Authors:** Alyt Damstra, Michael Hameleers

**Affiliations:** Amsterdam School of Communication Research, University of Amsterdam, Amsterdam, the Netherlands

**Keywords:** knowledge, media effects, crisis communication, negative information, panel surveys

## Abstract

This paper focuses on the accuracy of COVID-19-related knowledge during the 2020 pandemic. We look at the effects of traditional vs. digital news use and distinguish between positive (number of recoveries) and negative (number of casualties) knowledge. Importantly, the moderating role of crisis context is examined when tracing media effects on knowledge. Relying on a four-wave panel survey fielded in the Netherlands, we find that people’s knowledge became more accurate over the course of the crisis. News exposure did not lead to more accuracy, in fact, a negative relation was found. The impact of digital news use weakened as the crisis continued.

The new coronavirus (SARS-CoV-2) and the disease it causes (COVID-19) dominated media agendas across the globe in 2020. During this unfolding crisis, the relationship between the supply- and demand-side of information has been under pressure: people were in need of reliable information about the novel threat. While the media attempted to deliver this information, they also had to deal with high levels of uncertainty and expert disagreement. Over the course of the crisis, the availability of accurate information increased, as did information fatigue among the audience.

Against this backdrop, the acquisition of knowledge is not a given. Importantly, accurate knowledge may facilitate efficient policymaking and compliance. Over- or underestimating the consequences of the pandemic may result in behaviors not conducive to the alleviation of the crisis (i.e., hoarding). Misinformed citizens are, for example, found to be less likely to comply with instructions of the authorities (Hameleers et al., 2020). In this exploratory study, we examine (a) how the accuracy of knowledge related to COVID-19 developed over the course of the pandemic; (b) the impact of traditional vs. digital news use; and (c) whether and to what degree the acquisition of knowledge has been different for positive (recoveries) vs. negative (casualties) information. To answer these questions, we rely on a four-wave panel survey among a representative sample of Dutch citizens that was fielded between April and July 2020. The corona pandemic offers a unique case to examine how knowledge acquisition develops as a crisis situation changes in nature and severity. Theoretically, we aim to offer novel insights into the context dependency of knowledge acquisition. As disinformation tends to flourish in times of crisis (e.g., Pennycook et al., 2020), the acquisition of accurate information—crucial for democratic decision-making—may be hampered. Our findings show that it is crucial to take time into account: as the crisis context changes, the accuracy of people’s judgments may be affected by different factors.

## Knowledge acquisition across different stages of the outbreak

The concept of knowledge, although seemingly straightforward, has been plagued by conceptual disagreement (McKasy et al., 2020). Scholars often distinguish between denotative and connotative knowledge (Graber, 2001). Denotative knowledge is the most basic factual information that can be learned, such as statistical information. Connotative knowledge, however, refers to the integration of novel information with prior knowledge, and the process by which meaningful connections or schema are made between the two. In this paper, we focus on denotative knowledge by measuring the accuracy of people’s recall of number of deadly victims and recoveries from COVID-19. Taking into account that perceived and factual knowledge are oftentimes conflated (Su et al., 2014) and that people tend to overestimate their perceived knowledge (McKasy et al., 2020), factual estimates of consistently reported statistics during the pandemic may, in this case, offer the most valid operationalization of knowledge acquisition.

To trace the way in which knowledge developed during the pandemic, we must account for the changing nature of the crisis when the survey was fielded. The Netherlands represents a typical European country situated in the epicenter of the outbreak: the national numbers—related to new cases, deaths, and recoveries—reflect the European average. During the first stage (April), the pandemic claimed many victims and concerns were voiced about the capacity of intensive care units to treat patients. Despite expert disagreement—about virus spread and scope and most effective countermeasures—the government decided to implement strict interventions to slow down the spread of the virus. In the second phase (May), increasing expert consensus led to a higher availability of agreed-upon verified knowledge and facts. State interventions remained in force. The third stage (May/June) was characterized by significant drops in victims and increasing recovery rates. The media started to cover other issues alongside corona-related affairs. Finally, the fourth phase included in this study (June/July) is characterized by the gradual recovery of public life. Although the crisis’ alleviation caused optimism, concerns about “second waves” arose quickly as well.

During these months, two factors may have impacted people’s knowledge in contrasting ways. First, people’s factual knowledge may have increased as expert disagreement decreased (Dooren & Noordegraaf, 2020). Also, as in many countries, the recording of casualties and recoveries became more precise and reliable over time. At the same time, when the number of new cases went down, the saliency of the matter decreased. Having dominated public life in the Netherlands for months, information fatigue may have occurred among the audience, impeding processes of knowledge acquisition. In light of these contrasting perspectives, we raise the following exploratory research question (RQ_1_): How has the accuracy of people’s factual knowledge related to COVID-19 developed over time?

## Knowledge acquisition through media exposure: offline vs. online news

Extant research on knowledge acquisition has looked into the differences between offline vs. online news exposure (e.g., Eveland et al., 2004). Compared to traditional media outlets, digital information environments afford citizens more control, which also indicates that confirmation-biased selective exposure may be more likely to occur. In addition, consuming digital news requires more literacy: people must be able to navigate complex high-choice information settings. These affordances of digital information settings have, on the one hand, been associated with less knowledge acquisition (Bennett & Iyengar, 2008). Psychological and technological confirmation biases (Gil de Zúñiga et al., 2017) may impede learning from the news: algorithms may determine the information people are exposed to, and it requires critical media literacy skills to overcome the “trap” of these online confirmation biases.

Alternatively, it has been argued that digital information settings actually stimulate learning: people are motivated to find information that best fits their interests, and with the availability of high-speed Internet, exposure takes place whenever and wherever it suits them best (Lee & Ma, 2012). In addition, in digital information settings, citizens may come across different sources of information, and expose themselves to cross-cutting views from heterogenous networks (Mutz, 2002). Hence, they may not shut themselves off in partisan echo chambers but may learn from the other side instead. To explore these different dynamics, we pose the following research question (RQ_2_): How has the use of traditional and digital news sources affected the accuracy of people’s factual knowledge related to COVID-19?

To the best of our knowledge, no longitudinal research exists in which the relationship between news use and knowledge is examined across different stages of an unfolding crisis. Media dependency theory postulates that in a setting of high uncertainty, media become more important as people rely on them as first suppliers of information about the ongoing threat (Ball-Rokeach, 1985). Although it may be argued that uncertainty—based on a lack of empirical evidence and expert knowledge—was highest during the first stages of the pandemic, the overload of (conflicting) information during later stages could have cultivated uncertainty too. We therefore introduce a third research question: How has the impact of traditional and digital news exposure developed over time? (RQ_3_).

## Knowledge acquisition of positive vs. negative information

Our core-dependent variable, the accuracy of people’s factual knowledge related to COVID-19, consists of a negative and a positive dimension (estimates of casualties vs. estimates of recoveries). A rich body of work demonstrates how people, across a wide variety of settings, tend to be more responsive to negative compared to positive information (e.g., Baumeister et al., 2001). Also in the context of news effects, the asymmetric impact of tone is well-documented (e.g., Damstra & Boukes, 2018; Damstra et al., 2020). Based on this, one may anticipate that the impact of news use is strongest on people’s knowledge of casualties. The media’s tendency to highlight negative events, abundantly present during the first months of the pandemic, may further enhance this impact. On the other hand, positive information—such as news about people recovering from COVID-19—may become more salient in a crisis context as it lies further from the rather negative point of reference that people have.

In this study, no direct measures of news content were included, and therefore, we cannot assess diverging media effects for positive vs. negative content based on our multiwave panel data alone. However, we are able to examine how, over time, people’s estimates of casualties vs. recoveries develop and how both are affected by news exposure. To assess this, we formulate our fourth and final research question: To what extent do the impacts of time and media exposure differ across different types of knowledge (negative vs. positive)? (RQ_4_).

## Data and method

To answer our research questions, we rely on a four-wave panel survey conducted in the Netherlands, from April 10 to July 7, 2020. All materials, survey questions, and sampling techniques were preregistered before fielding the survey (Bakker et al., 2020). *I&O Research*, an ISO-certified research company, invited a representative sample of 3,750 Dutch adults, of which 1,742 completed the first questionnaire (completion rate: 46.0%). Only those respondents who participated in the previous wave were invited for the subsequent one. For Wave 2 (fielded from April 30 to May 11), 1,423 respondents completed the questionnaire (completion rate: 81.2%). In Wave 3 (May 25 to June 3), there were 1,241 completed questionnaires (completion rate: 87.2%) and 1,084 respondents participated in Wave 4 (June 29 to July 7; completion rate: 87.3%). The sample is reasonably representative of the Dutch population in terms of sex, age, education, and region, with a slight overrepresentation of higher educated and older respondents (see for a detailed overview the Supplementary Appendix). The mean attrition rate of 14.8% does not lead to significant changes in the distribution of demographic variables.

### Dependent variables

We measured factual knowledge by asking for estimates of the number of recoveries and casualties in the Netherlands. We decided to focus on these estimates as we aimed to distinguish between positive and negative knowledge. In addition, these statistics were consistently reported during the pandemic. For factual knowledge regarding casualties, people were asked to give an estimation of the number of people that, during the past 7 days, had passed away due to COVID-19 in the Netherlands. Similarly, for the number of recoveries, people were asked to give an estimation of the number of people that, during the past 7 days, had recovered from COVID-19 in the Netherlands. With regard to the latter question, recoveries were conceptualized as those people who had been hospitalized and that had left the hospital because of their recovery. To be clear, people did not have to give the exact correct number to be right: we looked at the discrepancy between real numbers and people’s estimates to assess levels of accuracy (i.e., lower discrepancy indicating higher accuracy). We thus deviate from approaches that let people rate statements as true or false (e.g., McKasy et al., 2020). Respondents with no numerical value (e.g., those who gave answers such as “a lot” or “not many”) were treated as missing.

In a next step, we calculated the correct answers for each day in which the survey was fielded. For the accurate number of casualties, we relied on figures provided by the National Institute for Public Health and the Environment, part of the Dutch Ministry of Health, Welfare, and Sport. For the accurate number of recoveries, we used figures released by the National Evaluation Intensive Care Foundation. The discrepancy between people’s estimates and the correct numbers was calculated straightforwardly by subtracting the correct numbers from the answers that people gave. As we are interested in the (in)accuracy of estimates and not in the specific direction (positive or negative) of inaccuracy, we converted all observations into positive values (higher values indicating more discrepancy between estimate and real number). Because both questions tapping into people’s knowledge were asked in an open-ended way, people were allowed to fill in their first association without being steered toward an answer category by the set-up of the survey item. This is a strength of the design, but it also makes the analyses prone to distortion by outliers. To adequately deal with this matter without having to determine arbitrary cutoff points, we standardized the variables and we excluded one observation with very extreme scores (estimates over a million).

### Independent variables

News use was measured by means of the question: “When thinking about last week, how many days have you watched/read/made use of [*specific news medium*]?” (scale ranged between 0 and 7 days). In order to measure the use of traditional news media, this question was asked for nine news programs broadcast on television and for six national newspapers. The sources differ in terms of signature and business model (quality/popular; public/commercial) but all sources belong to the Dutch mainstream media and aim to provide accurate and trustworthy information (see Supplementary Appendix Table SA2 for the complete list). Because our focus is on the frequency of news use, all items were summed in order to create an index indicative of traditional news use in terms of frequency. In a similar vein, we asked people how often they had used online news media. Five items tapped into the use of online news sites, which were summed to create an index indicative of online news exposure. In all analyses, we control for level of educational attainment (the ISCED categorization is split into three categories covering lower, medium, and higher educated citizens), governmental trust, gender, and age. To measure the impact of the crisis stage, we simply include the variable wave.

### Analytical approach

**Figure 1.**
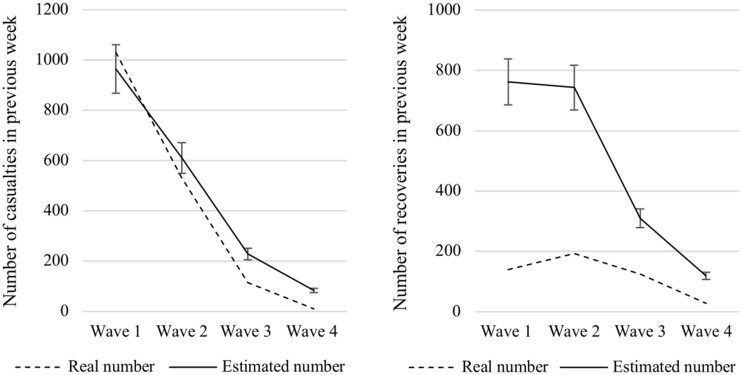
Casualties and recoveries: real and estimated numbers over time.

To analyze our data, we rely on regression models with lagged dependent variables and clustered standard errors (per respondent). The variables are standardized to rule out the possibility of effects being the result of the changing range of the variables (Figure 1). We used lagged values of trust and current values of news use (people were asked about their news use in the previous week). Education, age, and gender were treated as constant.

## Results

Figure 1 and Table 1 show how the accuracy of people’s estimates develop over time, presented alongside the average of real weekly numbers of casualties and recoveries. Respondents report highest estimates of people having passed away due to COVID-19 during the first waves of the survey, closely in line with real numbers. People’s estimates of recoveries are less accurate, as they consistently report more recoveries than there were in reality. The high standard deviations indicate substantial dispersion.

**Table 1.** Estimated casualties and recoveries per wave (mean and standard deviations).

	Wave 1	Wave 2	Wave 3	Wave 4
Casualties, *n*	1,684	1,389	1,209	1,058
Estimates, *M* (*SD*)	934 (828)	611 (756)	229 (803)	72 (560)
Inaccuracy, *M* (*SD*)	547 (626)	346 (677)	175 (792)	66 (560)
Recoveries, *n*	1,615	1,353	1,188	1,044
Estimates, *M* (*SD*)	698 (1,103)	672 (1,009)	310 (670)	108 (398)
Inaccuracy, *M* (*SD*)	598 (1,081)	525 (987)	232 (655)	88 (396)

Table 2 presents the regression models with lagged dependent variables, predicting the inaccuracy of knowledge related to casualties (Model 1) and recoveries (Model 2). We observe some interesting results that enable us to answer the research questions. First, knowledge becomes more accurate over time. The significant and negative effect of the wave variables indicates that the discrepancy between people’s estimates and real numbers decreases over the course of the crisis. Second, news use does not improve levels of accuracy. For knowledge related to casualties, using traditional news media leads to less accurate estimations, for knowledge related to recoveries, inaccuracy increases when people use digital news media. Third, the impact of digital news use on knowledge of recoveries is dependent on time. As the crisis continues, the effect weakens. Finally, impacts differ for estimates of casualties vs. recoveries. Knowledge of casualties is affected by traditional news use, while knowledge of recoveries is affected by digital news use. The significant lagged dependent variables in Model 1 indicate that past levels of knowledge predict current levels of knowledge, but only when related to casualties. Finally, we observe a gender difference but only for estimates of recoveries; female respondents tend to have more accurate knowledge.

**Table 2.** OLS regression explaining inaccuracy of knowledge—crisis stage and news exposure.

DV: Inaccuracy of factual knowledge				
	Model 1:		Model 2	
	Casualties of COVID-19		Recoveries from COVID-19	
	*b* (*SE*)	*b* (*SE*)	*b* (*SE*)	*b* (*SE*)
Constant	−0.01 (0.00)^***^	−0.01 (0.00)^***^	0.03 (0.01)^***^	0.03 (0.01)^***^
Lagged dependent variable	0.19 (0.06)^**^	0.19 (0.06)^**^	0.19 (0.11)	0.19 (0.11)
News use traditional	0.00 (0.00)^*^	0.00 (0.00)	−0.00 (0.00)	0.00 (0.01)
News use digital	−0.00 (0.00)	0.00 (0.00)	0.00 (0.00)^*^	0.02 (0.01)^**^
Traditional news use^*^wave		−0.00 (0.00)		−0.00 (0.00)
Digital news use^*^wave		−0.00 (0.00)		−0.01 (0.00)^**^
Governmental trust	−0.00 (0.00)	−0.00 (0.00)	0.00 (0.01)	0.00 (0.00)
Education	−0.00 (0.00)	−0.00 (0.00)	−0.00 (0.00)	−0.00 (0.00)
Age group	−0.00 (0.00)	−0.00 (0.00)	0.00 (0.00)	0.00 (0.00)
Gender (female)	−0.00 (0.00)	−0.00 (0.00)	−0.00 (0.00)^***^	−0.00 (0.00)^***^
Wave	−0.00 (0.00)^***^	−0.00 (0.00)^***^	−0.02 (0.03)^***^	−0.02 (0.00)^***^

Adjusted *R*^2^	0.07	0.07	0.08	0.08
*N*	3,583	3,583	3,458	3,458

* *p* < .05,

** *p* < .01,

*** *p* < .001 (two-tailed test).

## Discussion

Relying on Dutch panel data, this study explored knowledge acquisition at various stages of the 2020 pandemic. We distinguished between people’s knowledge related to casualties of COVID-19 and knowledge related to recoveries from COVID-19. As the crisis continued, knowledge increased. Especially regarding numbers of recoveries, people’s estimations became (much) more accurate over time. Different from what one might expect, the use of news media—traditional and digital—did not lead to more accurate knowledge. This could be related to the unprecedented circumstances under which this survey was fielded. The pandemic dominated public life for months and people may have been informed about the crisis through multiple channels. In addition, misinformation thrived online (Pennycook et al., 2020) and many of these stories were also (critically) covered by mainstream sources, which may have contributed to their dissemination (Tsfati et al., 2020). The negative effect of digital news use on the accuracy of knowledge about recoveries is most prominent during the first stages of the crisis. In a context of high uncertainty, the use of online sources may not contribute to knowledge.

Of course, this study is not without limitations. Relying on self-reported measures of news use is not a fine-grained strategy to capture news effects, which could explain the rather small effect sizes. In addition, our analyses do not reveal underlying mechanisms. Future research may look into the direction of the observed discrepancy, exploring whether news use leads to optimism or pessimism regarding the numbers. Our findings indicate that crisis context matters—directly as well as indirectly—and we hope to have provided a useful point of departure for future research into media effects under exceptional circumstances.

## Supplementary Data

Supplementary Data are available at *IJPOR* online.

## Supplementary Material

edab017_Supplementary_Data

## Notes

**Alyt Damstra** (PhD Communication Science, University of Amsterdam, 2020) is a postdoctoral researcher at the Amsterdam School of Communication Research, University of Amsterdam. Her research agenda includes media coverage and subsequent effects on political processes and public opinion. She is currently involved in a research project on knowledge resistance and works as a research fellow for the Scientific Council of Government Policy in The Hague.

**Michael Hameleers** (PhD, University of Amsterdam, 2017) is an Assistant Professor in Political Communication at the Amsterdam School of Communication Research (ASCoR), Amsterdam, the Netherlands. His research interests include framing, disinformation, populism, selective exposure, and attitudinal congruent persuasion.

## References

[edab017-B1] Bakker B. N. , van der Wal A., Vliegenthart R. (2020). COVID-19 panel study in the Netherlands. Amsterdam School of Communication Research, The Digital Communication Methods Lab and the Ministry of the Interior and Kingdom Relations. 10.17605/OSF.IO/KWZ7A

[edab017-B2] Ball-Rokeach S. J. (1985). The origins of individual media-system dependency: A sociological framework. Communication Research, 12(4), 485–510. 10.1177/009365085012004003

[edab017-B3] Baumeister R. F. , Bratslavsky E., Finkenauer C., Vohs K. D. (2001). Bad is stronger than good. Review of General Psychology, 5(4), 323–370. 10.1037/10892680.5.4.323

[edab017-B4] Bennett W. L. , Iyengar S. (2008). A new era of minimal effects? The changing foundations of political communication. Journal of Communication, 58(4), 707–731. 10.1111/j.1460-2466.2008.00410.x

[edab017-B5] Damstra A. , Boukes M. (2018). The economy, the news, and the public: A longitudinal study of the impact of economic news on economic evaluations and expectations. Communication Research. 48(1), 26-50. 10.1177/0093650217750971

[edab017-B6] Damstra A. , Boukes M., Vliegenthart R. (2020). To credit or to blame? The asymmetric impact of government responsibility in economic news. *International Journal of Public Opinion Research*, 33(1), 1–17. 10.1093/ijpor/edz054

[edab017-B7] Dooren W. V. , Noordegraaf M. (2020). Staging science: Authoritativeness and fragility of models and measurement in the Covid‐19 crisis. Public Administration Review, 80(4), 610–615. 10.1111/puar.13219 PMC7272834 32836435

[edab017-B8] Eveland K. Jr , Marton W. P., Seo M. (2004). Moving beyond “just the facts” the influence of online news on the content and structure of public affairs knowledge. Communication Research, 31(1), 82–108. 10.1177/0093650203260203

[edab017-B9] Gil de Zúñiga H. , Weeks B., Ardèvol-Abreu A. (2017). Effects of the news-finds-me perception in communication: Social media use implications for news seeking and learning about politics. Journal of Computer-Mediated Communication, 22(3), 105–123. 10.1111/jcc4.12185

[edab017-B10] Graber D. A. (2001). Processing politics: Learning from television in the Internet age. University of Chicago Press.

[edab017-B11] Hameleers M. , van der Meer T. G., Brosius A. (2020). Feeling “disinformed” lowers compliance with COVID-19 guidelines: Evidence from the US, UK, Netherlands and Germany. Harvard Kennedy School Misinformation Review, 1(3). 10.37016/mr 2020–023

[edab017-B12] Lee C. S. , Ma L. (2012). News sharing in social media: The effect of gratifications and prior experience. Computers in Human Behavior, 28(2), 331–339. 10.1037/t33075-000

[edab017-B13] McKasy M. , Cacciatore M., Su L. Y. F., Yeo S. K., Oneill L. (2020). Operationalizing science literacy: an experimental analysis of measurement. Journal of Science Communication, 19(4), A03. 10.22323/2.19040203

[edab017-B14] Mutz D. C. (2002). Cross-cutting social networks: Testing democratic theory in practice. American Political Science Review, 96(1), 111–126. 10.1017/s0003055402004264

[edab017-B15] Pennycook G. , McPhetres J., Zhang Y., Lu J. G., Rand D. G. (2020). Fighting COVID 19 misinformation on social media: Experimental evidence for a scalable accuracy nudge intervention. Psychological science, 31(7), 770–780. 10.31234/osf.io/uhbk9 32603243 PMC7366427

[edab017-B16] Su L. Y. F. , Cacciatore M. A., Scheufele D. A., Brossard D., Xenos M. A. (2014). Inequalities in scientific understanding: Differentiating between factual and perceived knowledge gaps. Science Communication, 36(3), 352–378. 10.1177/1075547014529093

[edab017-B17] Tsfati Y. , Boomgaarden H. G., Strömbäck J., Vliegenthart R., Damstra A., Lindgren E. (2020). Causes and consequences of mainstream media dissemination of fake news: Literature review and synthesis. Annals of the International Communication Association, 44(2), 157–173. 10.1080/23808985.2020.1759443

