# Supplementary material for: Knowledge Acquisition in Times of the 2020 Coronavirus Pandemic: Evidence from a Four-Wave Panel Study
Source: Int J Public Opin Res. 2021 Aug 30:edab017. doi: 10.1093/ijpor/edab017 (PMC8499763; doi:10.1093/ijpor/edab017)
Supplement: edab017_Supplementary_Data [file edab017_supplementary_data.docx]

Online appendix

*Table 1.* Demographic characteristics: sample per wave and Dutch population

|  | Sample | | | | Dutch population |
| --- | --- | --- | --- | --- | --- |
| Wave | 1 | 2 | 3 | 4 |  |
| Fielded | 10/04-20/04 | 30/04-11/05 | 25/05-03/06 | 29/06-07/07 |  |
| *N* | 1,742 | 1,423 | 1,241 | 1,084 |  |
| **Gender** |  |  |  |  |  |
| Male | 49.1 | 49.2 | 49.4 | 51.0 | 49.7 |
| Female | 50.9 | 50.8 | 50.6 | 49.0 | 50.3 |
| **Age group** |  |  |  |  |  |
| 18-39 | 31.5 | 29.9 | 30.9 | 29.6 | 33.3 |
| 40-64 | 44.3 | 44.2 | 42.7 | 43.0 | 33.7 |
| 65+ | 24.2 | 25.9 | 26.3 | 27.4 | 19.5 |
| **Education** |  |  |  |  |  |
| Low | 22.3 | 23.0 | 22.8 | 23.1 | 28.4 |
| Medium | 39.6 | 38.7 | 39.4 | 39.2 | 41.4 |
| High | 38.1 | 37.2 | 37.8 | 37.7 | 30.2 |
| **Region** |  |  |  |  |  |
| West | 42.9 | 42.6 | 42.0 | 42.0 | 45.6 |
| Nord | 11.1 | 10.8 | 10.4 | 9.8 | 9.9 |
| East | 22.3 | 22.2 | 22.1 | 22.1 | 21.1 |
| South | 23.6 | 24.5 | 25.5 | 26.1 | 23.3 |

*Note.* Population data are retrieved from Statistics Netherlands (CBS)

*Table 2.* Overview of media sources

| **Traditional news media** | |
| --- | --- |
| *NRC Handelsblad* | Newspaper |
| *De Volkskrant* | Newspaper |
| *Trouw* | Newspaper |
| *Algemeen Dagblad* | Newspaper |
| *De Telegraaf* | Newspaper |
| *Financieele Dagblad* | Newspaper |
| *NOS Journaal* | Television |
| *RTL Nieuws* | Television |
| *Hart van Nederland* | Television |
| *Editie NL* | Television |
| *M* | Television |
| *Nieuwsuur* | Television |
| *EenVandaag* | Television |
| *Op1* | Television |
| *Jinek/Beau* | Television |
| **Digital news media** | |
| Nos.nl | Website |
| Rtlnieuws.nl | Website |
| Nu.nl | Website |
| Rivm.nl | Website |
| Thuisarts.nl | Website |
